# Association of mental health help-seeking with mental health-related knowledge and stigma in Japan Rugby Top League players

**DOI:** 10.1371/journal.pone.0256125

**Published:** 2021-08-25

**Authors:** Yasutaka Ojio, Asami Matsunaga, Sosei Yamaguchi, Kensuke Hatakeyama, Shin Kawamura, Goro Yoshitani, Masanori Horiguchi, Shun Nakajima, Ayako Kanie, Masaru Horikoshi, Chiyo Fujii

**Affiliations:** 1 Department of Community Mental Health & Law, National Institute of Mental Health, National Center of Neurology and Psychiatry, Kodaira, Japan; 2 Japan Rugby Players’ Association, Japan; 3 National Center for Cognitive Behavioral Therapy and Research, National Center of Neurology and Psychiatry, Tokyo, Japan; St. Paul’s Hospital Millenium Medical College, ETHIOPIA

## Abstract

**Background:**

Globally increasing clinical and research interests are driving a movement to promote understanding and practice of mental health in elite athletes. However, few studies have yet addressed this issue. This study aims to describe the association of the intention to seek help with mental health knowledge and stigma and the severity of depressive symptoms in Japan Rugby Top League players.

**Methods:**

As a target population, we studied 233 Japan Rugby Top League male players (25–29 years = 123 [52.8%]), who were born in Japan, using a cross-sectional design. Surveys were conducted using anonymous, web-based self-administered questionnaires. Structural equation modelling was performed to evaluate the hypothesis of an interrelationship between mental health knowledge, stigma, and severity of depressive symptoms as factors influencing the intention to seek help.

**Results:**

Players with more severe depressive symptoms were more reluctant to seek help from others (β = - 0.20, p = 0.03). Players with greater knowledge about mental health tended to have less stigma toward others with mental health problems (β = 0.13, p = 0.049), but tended not to seek help with their own mental health problems.

**Conclusions:**

Rugby players in need of mental health support, even with greater knowledge, tend not to seek help from others, while having less stigma toward people with mental health problems. Rugby players might require approaches other than a knowledge-based educational approach to encourage them to seek help.

## Introduction

Mental health support for elite athletes, including rugby players, is a global issue. A certain prevalence (5% to 35%) of mental health problems in athletes has been reported by the International Olympic Committee (IOC) [1], and international expert consensus has shown that athletes need mental health support [1–7]. Globally, research into mental health problems and related behaviour in elite athletes has increased rapidly. The IOC Mental Health Working Group defines elite athletes as those who compete at a national or international level, including athletes in the domestic league [1].

Rugby society has been challenged to address this issue. World Rugby has documented the heavy load on physical and mental health associated with participating in competitive environments, and suggested that further research is needed to develop effective mental health support measures [8]. Professional rugby league players from the United Kingdom (UK) have exhibited mild (11.6%) or moderate/severe (2.6%) depressive symptoms, and likewise mild (18.9%) or moderate/severe (13.7%) anxiety symptoms [9]. We have reported comparable prevalence rates among Japan Rugby Top League players [10]. Despite the status among elite athletes and rugby players, they are often reluctant to seek help from others, resulting in a prolonged untreated period and inadequate early-stage intervention, according to expert opinion based on a narrative review [11]. As with previous studies in UK and Japanese general populations [12, 13], understanding the current status of mental health help-seeking and identifying relevant factors among players is crucial for exploring strategies to facilitate help-seeking in elite athletes.

To explore strategies for athletes to seek help with their mental health, we have considered previous research findings in the general population. Educational approaches based on a knowledge-attitude-behaviour model have been widely implemented [14, 15]. Mental health knowledge can help people recognize health problems, improve their attitudes to problems, and promote help-seeking [16]. A meta-analysis found that educational approaches and improvement in mental health knowledge were related to help-seeking for mental health problems in a general population [17]. In addition, according to the theory of mind [18], the intention to seek help is associated with helping others who have mental health problems. Those who reach out to people who have mental health problems indicate lower levels of mental health-related stigmatizing attitudes. Mental health first aid (MHFA) training programmes are expected to increase the intention to help others, and in a trial with young people, the intention to help others and seek help was promoted [19]. Several studies have shown that such MHFA intervention positively affects the coaches and staff around athletes [20, 21]. In short, promoting help-seeking in athletes might be achieved by increasing their mental health knowledge and reducing mental health-related stigmatizing attitudes, as in the general population.

Little is known about the empirical inter-relationship between knowledge of mental health, mental health-related stigmatizing attitudes, and the intention to seek help in elite athletes. A qualitative study [22] and a recent mixed-methods study [23] suggested a potential relationship between mental health knowledge and the intention to seek help among a sample of athletes. In particular, a recent study of rugby football league players in England and France [23] quantitatively addressed this issue and suggested that less knowledge about mental health support and greater embarrassment, pride, fear or shame were barriers to help-seeking. These findings supported review-based expert opinion that increasing mental health literacy encourages help-seeking behaviour in athletes [1–7]. However, multivariate statistical analysis to clarify the structural relationships with mental health help-seeking is needed. While previous studies have shown that multiple variables are associated with mental health help-seeking in elite athletes, few studies have quantitatively demonstrated structural relationships between these variables.

Mental health behaviour is known to be greatly influenced by national and regional cultural factors [24]. Due to a strong influence of stigma, people in the Asian ethnic group, including the Japanese, tend to hesitate more about seeking mental health treatment compared with populations from Western countries and other ethnic groups [25, 26]. However, research into this subject has been conducted and reported mainly in Australia, Europe, and the United States (US). To deepen international understanding of mental health help-seeking, evidence from different national and regional cultural settings is needed.

**Fig 1:**
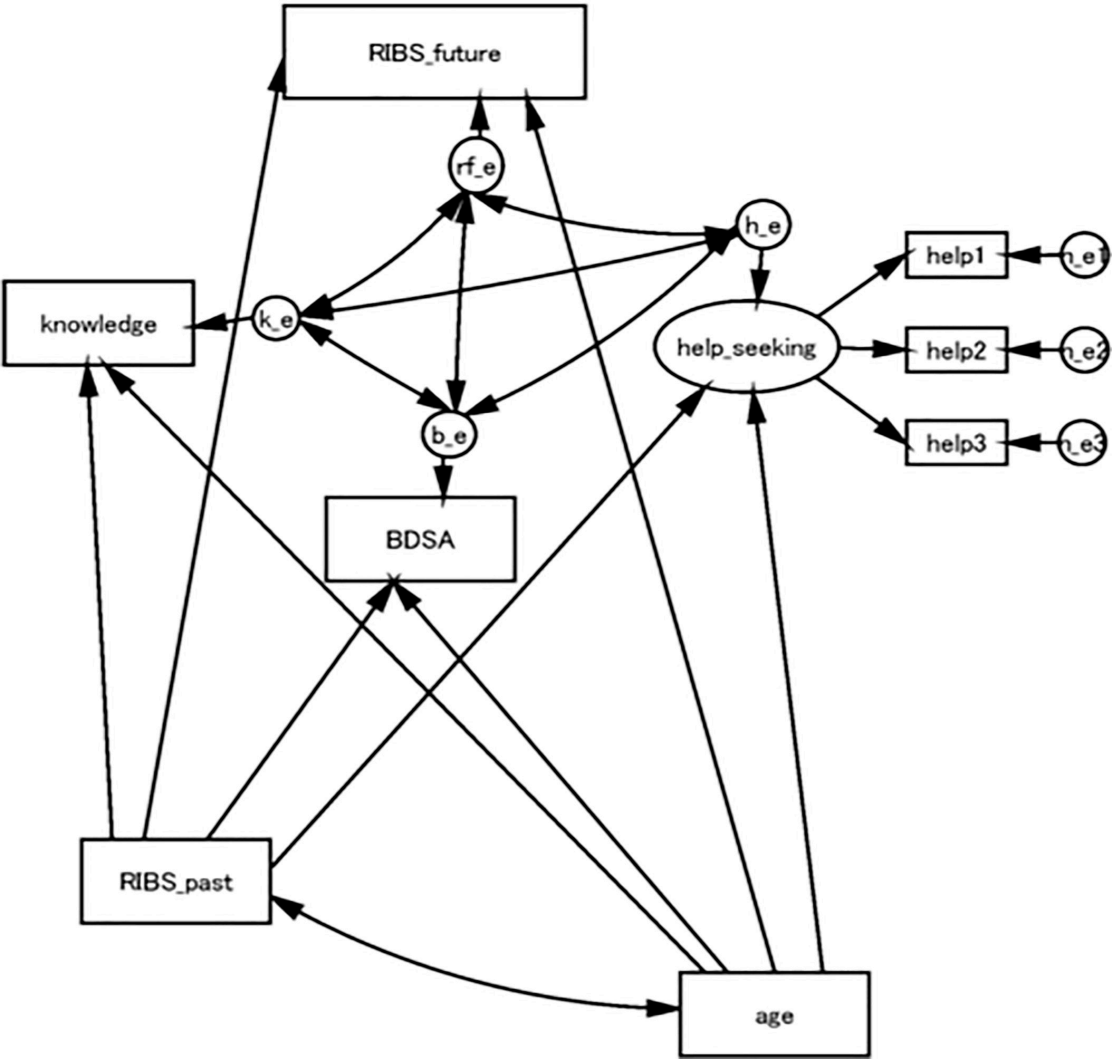
An initial structured equation model. Abbreviations: RIBS-J: The Japanese version of the Reported and Intended Behaviour Scale; BDSA: Baron Depression Screener for Athletes.

We have hypothesized a knowledge-attitude-behaviour model-based multiple pathway model of help-seeking intention, and a plausible multifactorial mechanism for seeking help, based on previous research (Fig 1). Poor knowledge of mental health reduces help-seeking because people do not recognize the symptoms of mental health problems and do not know where to seek help or support [16, 22, 27]. Those who experience mental health problems may avoid disclosing their problems or seeking help in order to avoid mental health-related stigma^,^ [22, 28]. Such stigma considerably reduces help-seeking and mental health service utilization, even with more severe symptoms of mental health status [29, 30]. Previous studies have suggested that these variables have a significant association with age [31, 32]. A path analysis with structural equation modelling (SEM) was performed to evaluate the hypothesis and to elucidate knowledge of mental health, mental health-related stigma, and age as factors in the intention to seek help. We have also examined the relationship between current mental health status and help-seeking intention by including the severity of depressive symptoms in this analysis. In the current study, we examine the interrelationship of each variable with help-seeking in Japan Rugby Top League players, a different national cultural sample from previous research.

## Materials and methods

### Study design and setting

We followed the STROBE guidelines for observational studies for the reporting of this cross-sectional study [33, 34]. The current study employed a web-based cross-sectional design. We distributed a survey URL to all players who were members of the Japan Rugby Players’ Association through team representatives participating in the regular meetings. All the players were invited to take an anonymous online survey, which did not include any identifiable variables, and which took about 10 minutes to complete. The participants were informed about the aim of the study, data collection procedures, and the implications of participation or non-participation in this study via the cover page of the questionnaire on the web. The participants were provided with individual one-time access by computer or tablet, using IP address filtering, to complete the survey. The funders had no role in the study design, data collection and analysis, decision to publish, or preparation of the manuscript. This study was approved by the Research Ethics Committee at the National Center of Neurology and Psychiatry (approval number: A2020-015).

### Patient and public involvement

No patients were involved in this study. Each participant received a summary of their findings. Members of the Japan Rugby Players’ Association were involved in the design, implementation, reporting and dissemination of our research.

### Participants

We collected data provided by 600 male rugby players, all aged 18 years and over, registered with the Japan Rugby Players’ Association. No exclusion criteria were applied. Out of this total, 251 participants gave consent (response rate: 39.1%). The response rate of this survey was not lower than other mental health surveys in Japan [35]. To minimize the influence of national cultural factors, of the 251 participants, we analysed 233 participants born in Japan as the target population in the current study. The remaining 18 players had been born in other countries and possibly not imbued in Japanese culture from an early age.

### Measures

In this study, we used a web survey to ask about knowledge of mental health, the Japanese version of the Reported and Intended Behaviour Scale (RIBS-J) for mental health-related stigma, and the Baron Depression Screener for Athletes (BDSA) for depressive symptoms, help-seeking intention scales, and demographic information. The components of the questionnaire are detailed below.

#### Knowledge of mental health

We developed items to evaluate mental health knowledge by modifying a verified and practically useful mental health knowledge questionnaire that had been used in past studies [36, 37]. A question about knowledge/recognition of specific symptoms and treatments was omitted. We also changed the wording of statements to make them more athlete-friendly. This section of the survey comprised six items (’True’/’False’/’Don’t know’): ’Mental illness affects 1 in 20 people during their lifetime’ (F), ’Mental illness is only for people who are weak and lacking guts’ (F), ’Leaving mental health issues untreated does not affect competitiveness’ (F), ’Mental illness is a brain disorder’ (T), ’Living environment (family, community, etc.) affects recovery from mental illness’ (T), and ’Mental illness is treatable’ (T). The answers were each scored: ‘Correct response’ = 1, ‘Incorrect’ or ‘Don’t know’ = 0. We calculated the total score of these answers and used this as the knowledge of mental health score. The total score ranged from 0–6.

#### RIBS-J

The RIBS-J consists of four binary items on past experience with people with mental health problems (RIBS-J past, range 0–4; ‘Yes’ = 1, ‘No’ = 0, ‘Don’t know’ = 0; higher scores represent more social contact), and four items on future behavioural intentions on a 5-point Likert scale (RIBS-J future, range 4–20; ‘Strongly agree’ = 5 to ‘Strongly disagree’ = 1; higher scores indicate a more positive intention, such as, ‘In the future, I would be willing to live with someone with a mental health problem’) [38, 39]. According to the RIBS-J development study, good model validity (e.g. comparative fit index (CFI) = 0.955 and root mean square error of approximation (RMSEA) = 0.07) and reasonable test–retest reliability (ρc = 0.71) of RIBS-J have also been reported [39]. In addition, Cronbach’s α was 0.59 and 0.78 in the present sample for the RIBS-J past and the RIBS-J future, respectively.

#### Help-seeking intention

We evaluated the intention to seek help with mental health problems by means of the following questions used in previous studies in Japan [13] and the UK [12]. First, whether the participants recognized the necessity to seek help from mental health professionals was assessed with the following question: ‘If you had a mental health problem, do you think it would be necessary to be supported by a professional?’ Responses were rated on a Likert scale of 1 to 5, with higher scores indicating a greater likelihood of recognition of the necessity to seek help. Second, whether participants had the intention to seek help from mental health professionals was measured by the following question: ‘If you felt that you had a mental health problem, how likely would you be to go to a mental health professional for help?’ Responses were rated on a Likert scale of 1 to 5, with higher scores indicating a greater likelihood of seeking help. The mean ± SD score for adults in the UK was 4.2 ± 1.1 [12] and for Japanese college students was 3.7 ± 0.9 [13]. Third, whether the participants felt comfortable disclosing a mental illness to friends or relatives was measured by the question: ‘In general, how comfortable would you feel talking to a friend or family member about your mental health, for example, telling them you have a mental health diagnosis and how it affects you?’ Scores ranged from 1 to 7, with higher scores reflecting greater comfort with disclosure. The mean ± SD score for the adults in the UK was 5.1 ± 1.9 [12] and for the Japanese college students was 3.1 ± 1.6 [13].

#### Baron depression screener for athletes

The BDSA is a 10-question self-report depression screening tool specifically for use with athletes [40]. The utilization of the BDSA for screening is recommended by the IOC’s consensus statement on mental health for athletes [1]. The BDSA addresses mood, sports-related anhedonia, weight loss, fatigue, self-image, substance abuse, suicidality, and other parameters over the past 2 weeks. It consists of 10 items on a three-point Likert scale (range 0 to 20, a higher score representing more severe depressive symptoms). We developed a Japanese version of BDSA (BDSA-J) and confirmed the one-factor structure of this scale to be the same as that of the original version of BDSA [41].

#### Statistical analysis

First, we calculated the mean, SD, skewness and kurtosis of all observed variables and correlation coefficients of these variables. Then we examined the hypothetical model using structural equation modelling (SEM) (Fig 1). Here we hypothesized that knowledge of mental health, RIBS-J future, BDSA, help-seeking intention were significantly associated with each other. In addition, past experience of contact with people with mental illness and age would affect variables in the model. We assumed these to be control variables and added paths from RIBS-J past or age to knowledge, RIBS-J, BDSA, and help-seeking, respectively. Knowledge, RIBS-J future, RIBS-J past, BDSA and age were used as observed variables. Since each question about help-seeking had different scoring, we created latent help-seeking variables using three indicators of help-seeking. As the causal relationships among knowledge, RIBS-J future, help-seeking, and BDSA could not be examined in this cross-sectional study, we assumed covariance between each pair of these variables in the model. The estimation of the model was conducted using maximum likelihood estimation. The fit of the model with the data was examined in terms of chi-squared (CMIN), comparative fit index, and root mean square error of approximation. According to conventional criteria, a good fit would be indicated by CMIN/df<2, CFI>0.97, and RMSEA<0.05, while CMIN/df<3, CFI>0.95, and RMSEA<0.08 demonstrate an acceptable fit [42]. All statistical analyses were conducted using Stata version 16 and Amos 25.

## Results

### Descriptive data

Demographic variables of the study participants are shown in Table 1. Over half of the participants (52.3%) were 25–29 years old, and 97.5% had graduate university educational attainment. 48.1% of them were married and 28.9% had a child in the family. About half of them lived with family or a partner. 19.2% of them had experience on the national team and 37.8% reported that they had not played in a competition in the last season.

**Table 1:** Demographic and knowledge-attitude-behaviour related variables of the study participants.

	% (n)
Age at survey	
19~24	20.2 (47)
25~29	52.3 (123)
30~34	24.7 (58)
35~	3.0 (7)
Educational attainment	
High school	0.85 (2)
Four-year college or university	97.5 (229)
Postgraduate college (or higher)	1.7 (4)
Marital status	
Married	48.1(113)
Never married	51.1 (120)
Divorced or widowed	0.9 (2)
Residential Status	
Living alone	17.5 (41)
Living with family and/or partner	50.6 (119)
Dormitory	31.9 (75)
Child living in household, Yes	28.9 (68)
Experience of national team, Yes	19.2 (45)
Playing status of last season	
As a active member	29.8 (70)
As a reserve member	32.3 (76)
No play	37.9 (89)

### Outcome data

Correlation coefficients, mean, SD, skewness and kurtosis of the knowledge-attitude-behaviour variables and ages, used in the model are shown in Table 2, and univariate normality was observed.

**Table 2:** Pearson’s correlation coefficients, mean, SD, skewness and kurtosis of observed variables.

		1	2	3	4	5	6	7	8
1	Knowledge of mental health	-							
2	RIBS-past	0.036	-						
3	RIBS-future	0.132*	0.214**	-					
4	help-seeking 1: intention to seek help	0.119	-0.078	-0.009	-				
5	help-seeking 2: intention to use mental health services	0.083	-0.041	-0.035	0.525***	-			
6	help-seeking 3: intention to disclose	-0.010	-0.031	0.096	0.069	0.206**	-		
7	BDSA	-0.018	0.022	0.060	-0.108	-0.179**	-0.143*	-	
8	age	0.034	0.071	-0.066	0.095	0.105	-0.054	-0.029	-
	mean	3.811	0.670	12.893	4.107	4.004	4.146	5.017	3.094
	(SD)	(1.033)	(0.986)	(3.198)	(0.938)	(0.812)	(1.830)	(3.034)	(0.754)
	skewness	-0.885	1.510	-0.082	-1.281	-0.976	-0.195	0.335	0.206
	kurtosis	4.679	4.577	3.462	4.610	4.410	2.164	2.759	2.881

RIBS-J, The Japanese version of the Reported and Intended Behaviour Scale; BDSA, Baron Depression Screener for Athletes

* p < .05

** p < .01

*** p < .001

### Main results

**Fig 2:**
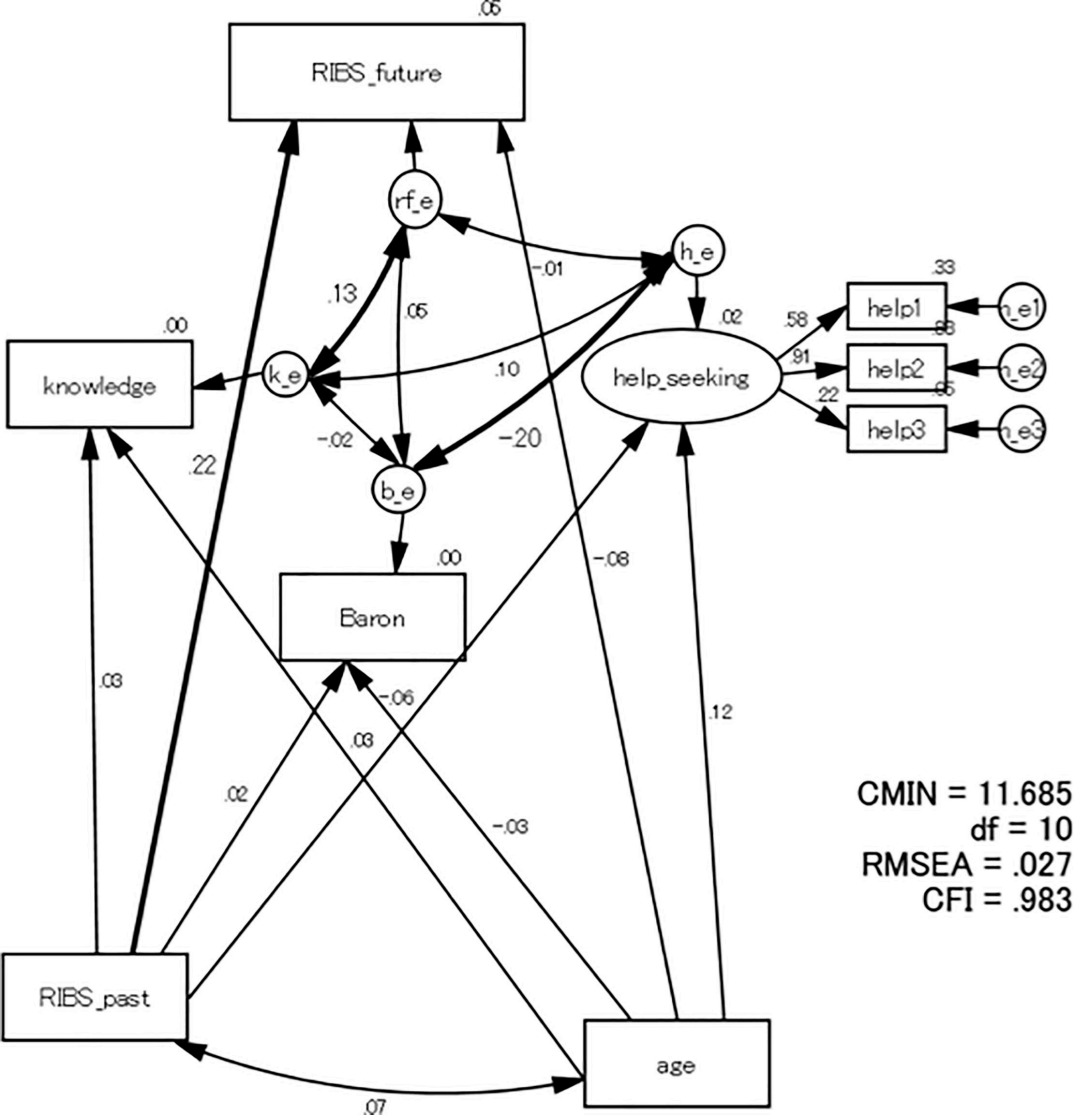
Results of the structured equation model. Abbreviations: RIBS-J: The Japanese version of the Reported and Intended Behaviour Scale; BDSA: Baron Depression Screener for Athletes.

The results of SEM are shown in Fig 2. The model shows a good fit: χ^2^ (CMIN) = 11.685, df = 10, CMIN/df = 1.169 (p = 0.307), CFI = .983, RMSEA = .027 (90%CI = .000-.079). Age had no statistically significant impact on any variable, including knowledge-attitude-behaviour intention. Only the BDSA score was directly associated with the intention to seek help (β = - 0.20, p = 0.03), indicating that the more severe the depressive symptoms, the less the intention was to seek help from others. Neither knowledge of mental health nor RIBS-J future predicted help-seeking intention (p > 0.05). RIBS-J future was predicted by RIBS-J past (β = 0.22, p < .001) or associated with knowledge of mental health (β = 0.13, p = 0.049). This in turn demonstrated that both more experience of social contact with people with mental health problems and higher levels of knowledge of mental health were associated with a more positive future attitude towards people with mental health problems. The associations between knowledge and help-seeking, knowledge and BDSA, RIBS-J future and help-seeking, and RIBS-J future and BDSA, respectively, were not significant.

## Discussion

The present study has examined the association of the intention to seek help, mental health knowledge and stigma, and severity of depressive symptoms in Japan Rugby Top League players, to achieve a greater understanding of potential factors in mental health help-seeking. To clarify factors associated with help-seeking and their structural relationships in elite athletes not considered in previous research, we have referred to a knowledge-attitude-behaviour model used in the general population [14, 15]. The current results demonstrate that players in need of mental health support tend not to seek help with their problems, even with greater knowledge of mental health. This is here demonstrated in the mental health-related behaviour of Japan Rugby Top League players.

The Japan Rugby Top League players with more severe depressive symptoms were more reluctant to seek help from others. This may possibly indicate a bidirectional relationship: lack of seeking help and receiving support may lead to poor mental health, while people with poor mental health are less likely to have the opportunity to receive the necessary support. These results are consistent with previous academic knowledge and clinical experience of a general population [43], which may be replicated in athletes, including rugby players [44, 45]. Based on expert opinion [46, 47], the fact that players in need of mental health support tend not to seek help or receive support with their problems should be shared among players, their coaches, health professionals, and others who work with players.

In the players, knowledge of mental health was not associated with the intention to seek help, and this association was also not affected by past experience of social contact with people who had mental health problems. This finding is contrary to our hypothesis based on the findings in the general population. These findings may stem largely from the individual or cluster’s characteristics in the current sample of athletes. The review paper [45] has pointed out that traditional masculine gender ideals such as stoicism, self-reliance and restrictive emotionality, in addition to mental health-related knowledge and past experience, are significant barriers to seeking help for athletes, including rugby players. When players themselves experience mental health problems, they tend to refuse to recognize or display the problems, because they fear being perceived as weak or non-masculine by those around them or by the general public [48]. Rugby players may have high traditional masculinity, and they are in a competitive sports society where great stoicism is required [49, 50]. Normative masculine ideals in interpersonal, social, cultural and structural factors embedded in the society may have negative effects on help-seeking barriers for rugby players. In such communities, improving knowledge may be unlikely to be related to the level of help-seeking. The findings may also be relevant to health behaviour in other male-dominated communities, such as the military, business management, and politics [51].

We found that players with greater knowledge about mental health have a less stigmatizing attitude toward people with mental health problems. The relationships between these variables are inconsistent with previous studies, which vary between the study population types. Health professionals, who have greater knowledge than the general population, often have more negative attitudes toward mental illnesses [52, 53]. In a general population, a meta-analysis reported that more knowledge of biological correlates of mental illness was associated with a greater degree of stigmatizing attitudes to schizophrenia [54]. But this was not found to be the case with regard to depression [55]. Other studies in general populations showed a positive correlation between these variables, i.e., more knowledge was associated with better attitudes [56, 57]. The rugby players who participated in the current study may have been subject to group-specific factors. For example, positive aspects of normative masculine ideals, such as a sense of social justice and a willingness to help those in need, may have had an impact on this relationship. Our finding in the population of rugby players suggests that knowledge improvement may be associated with a less stigmatizing attitudes towards others experiencing mental health problems.

We have demonstrated several factors that promote (or hinder) the intention to seek help in rugby players, but were not able to identify other possible factors because the knowledge-attitude-behaviour model did not include relationships with social and environmental factors. The theory of planned behaviour, which is a more developed health behaviour theory, might predict an individual’s behaviour through their intention to perform the actual behaviour [58]. This behavioural intention is influenced by three factors: attitudes, subjective norms, and perceived behavioural control. Concerns including high traditional masculinity and a highly competitive sports society that requires a great degree of stoicism, which are closely related to the elite athlete population, specifically correspond to "subjective norms". In addition, the variables of self-efficacy contained in "perceived control" facilitate an understanding of help-seeking intentions in elite athletes.

### Strength and limitations of this study

To the best of our knowledge, this is the first study to investigate mental health behaviour and relevant factors among elite athletes in Japan. The novelty of the current study lies in the new findings from Asia, which differ from those in Australasia, the US, and the UK, where most previous studies on athletes’ mental health have been conducted.

We recognize several limitations that should be taken into consideration. First, the current sample was male rugby players in the Japan Rugby Top League. In future studies, replication research should be conducted in other countries, other sports, and with female athletes. Given that there may be major cultural differences in views of mental health-related behaviour in different countries or regions [25, 26, 59], studies in various countries are required. Second, while the response rate to this survey is comparable to other mental health surveys [35], it is not sufficient to accurately estimate Japanese elite athletes’ overall trend. A specific challenge in mental health surveys is that people who have mental health-related stigma and more severe symptoms may not be willing to respond or to engage in such surveys [60]. Third, the items relating to mental health knowledge included in this study may not be sufficient. For example, ’mental health literacy’ focusing on providing information about when and where to seek help may be considered to an important element [61, 62]. Therefore, practically useful knowledge, which we did not include in our survey, might impact the degree of intention to seek help. Fourth, since the data were obtained by a cross-sectional design and were insufficient to infer the existence of a causal relationship, we have provided an interpretation of the phenomenon suggested by the findings of previous studies. To verify this, a longitudinal study, using lifelogs, and/or intervention studies with a conditioned control group would be needed.

## Conclusions

While globally increasing clinical and research interests are driving a movement to promote and support mental health for elite athletes, only a small number of studies have addressed this issue. We have assessed mental health status, and knowledge, attitudes, and behaviour related to mental health in Japan Rugby Top League players. The study indicates a significant association between severe depressive symptoms and more reluctance to seek help from others. However, we found neither significant associations between mental health knowledge and help-seeking intentions, nor between social contact experience and mental health knowledge or help-seeking intentions. Based on these findings, interventions which do not focus on knowledge-based educational approaches might be required. Further studies having other samples and other potentially relevant variables are needed to confirm the findings.

## Supporting information

S1 Checklist (DOCX)
